# *Lactobacillus plantarum* and *Lactobacillus brevis* alleviate enterotoxigenic *Escherichia coli*-induced intestinal inflammation and stabilize intestinal microorganisms and serum metabolites in piglets

**DOI:** 10.1016/j.aninu.2025.04.005

**Published:** 2025-06-20

**Authors:** Xuebing Han, Rong Gao, Sujuan Ding, Hao Yao, Jun Fang, Gang Liu

**Affiliations:** a College of Bioscience and Biotechnology, Hunan Agricultural University, Hunan Provincial Engineering Research Center of Applied Microbial Resources Development for Livestock and Poultry, Changsha, Hunan 410128, China; b Changsha IMADEK Intelligent Technology Co., Ltd., Changsha, Hunan 410000, China

**Keywords:** *Lactobacillus plantarum*, *Lactobacillus brevis*, Enterotoxigenic *Escherichia coli*, Metabolite, Intestinal microorganism, Transcriptome

## Abstract

Enterotoxigenic *Escherichia coli* (ETEC) is a major pathogen causing diarrhea in weaned piglets, and oral administration of probiotics has become an important strategy for preventing and treating ETEC-induced diarrhea. The aim of this study was to investigate the protective effect of two probiotics, *Lactobacillus plantarum* and *Lactobacillus brevis*, on ETEC-induced injury in piglets. Twenty-four piglets were randomly divided into control (CON) group (fed with a basal diet and treated with sterile saline), ETEC group (fed with the basal diet and treated with ETEC), LP-ETEC group (fed with the basal diet and treated with ETEC and *L*. *plantarum*) and LB-ETEC group (fed with the basal diet and treated with ETEC and *L. brevis*), with 6 piglets in each group. The experiment lasted for 21 days. The results indicated that both probiotics were able to inhibit the ETEC-induced reduction in daily weight gain of piglets and prevent the damage to jejunum. *L*. *brevis* significantly increased daily weight gain (*P* = 0.012), while *L*. *plantarum* showed a similar trend (*P* = 0.063). Serum metabolomic analysis showed that both probiotics significantly increased some beneficial metabolites including phosphatidylethanolamine, monoacylglyceride, cholic acid and rhodoxanthin (*P* < 0.05). Transcriptome sequencing indicated that *L. plantarum* and *L. brevis* may alleviate piglet diarrhea by regulating genes including *LBH* and *RNASE1*. According to 16s rRNA sequencing, both probiotics were able to maintain the normal microbial community of the gut. In summary, *L. plantarum* and *L. brevis* can protect piglets from ETEC-induced damage and promote healthy growth of piglets by regulating metabolites and gene expression.

## 1 Introduction

Diarrhea, a significant global health challenge, is a leading cause of mortality not only among children under the age of 5 in developing countries but also in piglets (Chavda et al., 2024). It can be transmitted from asymptomatic carrier sows or diarrheic piglets to naive animals (Dubreuil et al., 2016). Enterotoxigenic *Escherichia coli* (ETEC) is the most common foodborne epidemic pathogen that causes diarrhea (von Mentzer and Svennerholm, 2024). The adhesins and enterotoxins produced by ETEC promote the attachment of pathogens to the intestinal epithelium of piglets, leading to fluid-electrolyte disturbance and acid-base imbalance (Dubreuil, 2017; Joffré et al., 2016). After attachment to the intestinal epithelium, ETEC releases heat-labile enterotoxins (LT) and/or heat-stable enterotoxins (ST), which disrupt electrolyte homeostasis in the intestinal epithelium, causing fluid loss and ultimately leading to diarrhea (Kaper et al., 2004). Diarrhea causes disruption of the intestinal flora, triggering the dysregulation of multiple physiological functions in the host, such as digestion, metabolism, and immunity (Li et al., 2021). Thus, it is crucial to find suitable feed supplements that can suppress the proliferation of pathogenic bacteria, maintain intestinal health, and prevent intestinal damage caused by diarrhea during piglet growth.

Probiotics are defined as “live microorganisms that, when administered in adequate amounts, confer a health benefit on the host” (Cheng and Ouwehand, 2020). In animals, probiotics can serve as feed supplements to provide benefits to the host through a range of mechanisms, including inhibition of pathogen adhesion and colonization in the animal gut, reduction of bacterial translocation, and enhancement of the barrier function of the gut through interaction with the intestinal epithelium and immunocompetent cells, and production of a variety of substances with antibacterial effects, including organic acids, hydrogen peroxide and bacteriocins (Filidou et al., 2024; Kim et al., 2019). Most probiotics are derived from the intestinal tract and belong to lactic acid bacteria. Among these, *Lactobacillus plantarum* and *Lactobacillus brevis* are promising and effective feed additives that can promote the production of unsaturated fatty acids, accelerate fat metabolism, inhibit the proliferation of harmful bacteria, maintain the balance of the intestinal environment and reduce intestinal inflammation (Fuke et al., 2018; Han et al., 2021; Wang et al., 2018). Based on the known antimicrobial properties of probiotics, we hypothesized that dietary supplementation with *L. plantarum* and *L. brevis* alleviates ETEC-induced diarrhea in piglets by restoring intestinal microbiota homeostasis to enhance barrier function, and modulating host metabolic pathways related inflammatory responses.

The effects of two probiotics, *L. plantarum* and *L. brevis*, on intestinal morphology in piglets infected with ETEC were investigated, and the changes in intestinal microbiota, body metabolism, and gene expression by high-throughput sequencing, metabolomics, and transcriptome sequencing were analyzed in this study, with the purpose is to provide new ideas for exploring the interaction mechanism between probiotics and the body, and provide a theoretical basis for using probiotics to prevent diarrhea in neonatal animals.

## 2 Materials and methods

### 2.1 Animal ethics statement

The experiment was approved by the Animal Care and Use Committee of Hunan Agricultural University (Approval No. 202033).

### 2.2 Bacteria culture

The strains L. *plantarum* and *L*. *brevis* used in this study were isolated and preserved by our group at Hunan Agricultural University (Changsha, China), and grown in De Man, Rogosa and Sharp medium broth (Solarbio, M8540, storage at room temperature). The ETEC K88 strain (serotype O149:K91:K88ac), obtained from the China Institute of Veterinary Drug Control (Beijing, China), was grown in Luria-Bertan media (Solarbio, L1010, storage at room temperature). For colony quantification, the bacterial fluid that had been incubated for 24 h was transferred to the appropriate solid medium and then cultured for another 24 h at 37 °C. After that, both *L*. *plantarum* and *L*. *brevis* were suspended in sterile saline at a concentration of 2 × 10^10^ to 5 × 10^10^ CFU/mL, and ETEC at 1 × 10^9^ CFU/mL.

### 2.3 Animal experiment design

**Fig. 1:**
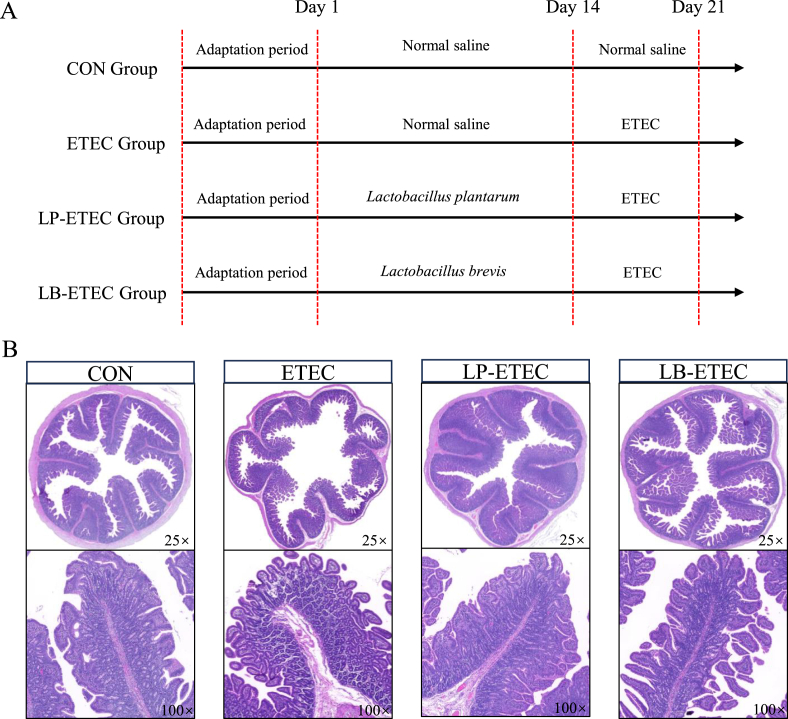
Experimental process and jejunal tissue damage of piglets. (A) Experimental process. (B) Jejunal tissue hematoxylin and eosin (H&E) staining. CON: treated with sterile saline; ETEC: treated with *E. coli*; LP-ETEC: treated with ETEC and *L. plantarum*; LB-ETEC: treated with ETEC and *L. brevis*.

Twenty-four weaned male piglets with an average weight of 7.28 ± 0.85 kg were randomly assigned to four groups: the control group (CON), ETEC-treated group (ETEC), *L. plantarum*-treated group (LP-ETEC) and *L. brevis*-treated group (LB-ETEC), with six piglets in each group. The pens with plastic mesh flooring (1 animal per pen) were placed in air-conditioned rooms to maintain a controlled humidity and temperature (30 ± 1 °C). Feed provision (08:00, 12:00, 16:00 and 20:00, four times a day) was sufficient, and the piglets were allowed free access to the diet. Firstly, they were acclimated in an ETEC-free environment for 5 days and then entered a 21-day trial period (Fig. 1A). Table 1 presents the ingredients and nutrient levels of the basal diet. The measurement methods for analyzing the contents of crude protein (GB/T 6432-2018), crude fiber (CF; GB/T 6434-2022), Ca (GB/T 6436-2018), P (GB/T 6437-2018), and amino acids (GB/T 18246-2019) in the diet were all based on the China National Standards. All diets were designed to fulfill the nutrient specifications outlined by the (NRC, 2012). The feed intake and body weight of piglets were recorded.

**Table 1:** Ingredients and nutrient levels of the basal diet (air-dried basis,%).

Item	Content
**Ingredients**	
Corn	57.80
Wheat bran	6.00
Fish meal	3.00
Soybean oil	2.00
Soybean meal	11.90
Soy protein concentrate	6.00
Fermented soybean meal	5.00
Whey power	6.00
Phytase	0.02
NaCl	0.30
Limestone	0.50
L-Lysine HCl	0.52
DL-Methionine	0.16
L-Threonine	0.16
L-Tryptophan	0.04
Premix^1^	0.50
Chloride choline	0.10
Total	100.00
**Nutrient levels**^2^	
Digestible energy, MJ/kg	25.76
Crude protein	18.27
Crud fiber	2.31
Calcium	0.79
Total phosphate	0.72
Available phosphorus	0.61
Lysine	1.22
Methionine	0.36
Threonine	0.76
Tryptophan	0.25

1 Premix provided the following per kg of the basal diet: vitamin A 12,000 IU, vitamin D_3_ 2500 IU, vitamin E 30 IU, vitamin K_3_ 3.0 mg, vitamin B_12_ 0.012 mg, riboflavin 4.0 mg, pantothenic acid 15.0 mg, niacin 40.0 mg, choline chloride 400.0 mg, folic acid 0.7 mg, vitamin B_1_ 1.5 mg, vitamin B_6_ 3 mg, biotin 0.1 mg, Mn 40.0 mg, Fe 90.0 mg, Zn 100.0 mg, Cu 8.8 mg, I 0.35 mg, Se 0.3 mg.

2 All the values in the nutrient composition are measured except for the calculated values of digestible energy (NRC, 2012). Digestible energy was calculated using data provided by the Feed Database in China (2019).

Within 1 to 14 days, piglets in LP-ETEC and LB-ETEC groups were fed diets containing *L. plantarum* and *L. brevis* at a concentration of 1.5 × 10^10^ CFU/kg per day, respectively (Lähteinen et al., 2014; Yang et al., 2014). The piglets in CON and ETEC groups were given sterile saline daily. After 14 days, piglets in the CON group were gavaged with 0.1 mL sterile saline once every three days, while 0.1 mL ETEC with a concentration of 1 × 10^9^ CFU/mL was given to the other three groups. Animals were checked daily for fecal consistency to evaluate their status after being challenged with ETEC. According to Liu's classification criteria (Liu et al., 2010), fecal consistency was visually evaluated and divided into four grades: 0, normal; 1, pasty; 2, semiliquid; and 3, liquid. When their fecal consistency fell into levels 2 or 3, piglets were considered to have diarrhea. The diarrhea incidence was determined by calculating the percentage of diarrhea days in each pen out of the total number of recorded days. On day 21, blood was drawn and all piglets were euthanized. Serum was separated, a portion of the jejunum was fixed, and colon contents were gathered for metabolomic analysis, histomorphometric analysis, microbial diversity analysis, and RNA-Seq, respectively.

### 2.4 Hematoxylin-eosin staining

Jejunal tissue samples of piglets were fixed in 4% paraformaldehyde. Then, the tissue samples were dehydrated and embedded in paraffin using graded concentrations of ethanol and xylene. Staining with hematoxylin and eosin was followed by observation using a microscope. The specific procedure was based on the method described in a previous study (Ma et al., 2020).

### 2.5 Analysis of serum metabolomics

The serum of piglets was obtained through ultrasonication. Subsequently, Liquid Chromatograph-Mass Spectrometer analysis was conducted in both positive-ion and negative-ion modes with voltages of 4.0 and 3.5 kV, respectively. The data after off-line were subjected to peak identification and peak comparison, and the data were statistically processed. The specific methods were based on a previous study (Han et al., 2023).

### 2.6 Whole transcriptome resequencing

As described previously (Zhang and Guo, 2009), total RNA was isolated from colon tissue samples of piglets by using TRIzol reagent (Invitrogen, Carlsbad, CA, USA). The whole transcriptome resequencing was completed by Beijing Biomarker Technologies Co., Ltd., Beijing, China. RNA integrity was evaluated and RNA purification, library construction and paired-end sequencing were performed based on the Illumina sequencing platform. After the expression level was standardized, DESeq was used to analyze the differences in gene expression (Love et al., 2014). The conditions for screening differentially expressed genes were as follows: |log_2_ (fold change [FC]) | > 1 and *P* < 0.05.

### 2.7 16S ribosomal RNA amplicon sequencing

After extracting the microbial genomic DNA from the colon contents of piglets, its concentration and purity were tested. Primers pair 357F (5′-ACTCCTACGGRAGGCAGCAG-3′) and 806R (5′- GGACTACHVGGGTWTCTAAT-3′) were used to amplify the V3–V4 region of the 16S rRNA. Then, the PCR products were recovered and purified. Finally, the operational taxonomic units clustering was used to control and optimize the quality of raw data and determine the microbial community, utilizing the mothur software (version 1.33.3) to perform the analysis of microbial α diversity.

### 2.8 Data analysis

The differences among groups were compared using the SPSS software version 21 (SPSS Inc., Chicago, IL, USA) through one-way ANOVA with Tukey's HSD. The data were presented as mean values ± standard deviation (means ± SD). The difference between groups was considered statistically significant when *P* < 0.05.

## 3 Results

### 3.1 Probiotics alleviate ETEC-induced growth inhibition

According to the results in Table 2, compared to the CON group, the average daily weight gain of piglets in the ETEC group was reduced. From day 1 to 20, *L. brevis* significantly increased the average daily weight gain of piglets (*P* = 0.012), *L. plantarum* also elevated the average daily weight gain but didn't show the significance (*P* = 0.063). Additionally, both probiotics significantly reduced the F:G (*P* < 0.05). Moreover, *L. plantarum* and *L. brevis* significantly decreased the ETEC- induced diarrhea incidence in piglets (*P* < 0.001).

**Table 2:** The growth performance and diarrhea incidence of piglets.^1^

Item	CON	ETEC	LP-ETEC	LB-ETEC	*P* -value (ETEC vs LP-ETEC)	*P*-value (ETEC vs LB-ETEC)
**BW, kg**						
Day 1	7.66 ± 0.77	7.33 ± 0.91	7.02 ± 0.87	7.10 ± 0.55	0.925	0.969
Day 14	13.60 ± 0.77	13.01 ± 0.64	13.20 ± 0.89	13.45 ± 0.61	0.979	0.783
Day 20	17.29 ± 1.15	15.67 ± 0.80	16.63 ± 0.99	17.09 ± 0.99	0.430	0.136
BW difference, kg	9.63 ± 0.69 ^ab^	8.34 ± 0.84^b^	9.62 ± 0.82 ^ab^	9.99 ± 0.63^a^	0.063	0.012
**Day 1–14 (pre-challenge)**						
ADG, g	424.52 ± 17.29	406.31 ± 35.26	441.43 ± 29.18	453.69 ± 18.12	0.349	0.132
ADFI, g	592.17 ± 57.65	536.72 ± 28.29	582.11 ± 55.87	589.02 ± 25.71	0.393	0.276
F:G	1.40 ± 0.07	1.33 ± 0.13	1.32 ± 0.06	1.30 ± 0.05	0.994	0.938
**Day 15–20 (*E. coli* challenge)**						
ADG, g	615.00 ± 70.53^a^	442.22 ± 89.73^b^	572.78 ± 73.53 ^ab^	606.67 ± 75.77^a^	0.067	0.016
ADFI, g	886.76 ± 61.43	844.26 ± 68.41	894.18 ± 87.12	920.16 ± 71.19	0.702	0.374
F:G	1.46 ± 0.14^b^	1.96 ± 0.30^a^	1.57 ± 0.09^b^	1.53 ± 0.09^b^	0.011	0.005
Diarrhea incidence,%	8.33^c^	66.67^a^	30.56^b^	27.78^b^	<0.001	<0.001
**Day 1–20 (pre- and *E. coli* challenge)**						
ADG, g	481.67 ± 34.54 ^ab^	417.08 ± 42.19^b^	480.83 ± 40.84 ^ab^	499.58 ± 31.41^a^	0.063	0.012
ADFI, g	680.55 ± 58.05	628.98 ± 35.41	675.73 ± 60.26	688.36 ± 36.54	0.451	0.253
F:G ratio	1.41 ± 0.03^b^	1.52 ± 0.10^a^	1.41 ± 0.04^b^	1.38 ± 0.03^b^	0.032	0.007

BW = body weight; ADFI = average daily feed intake; ADG = average daily gain; F:G = the ratio of feed intake to weight gain.

The absence of identical letter marker indicated significant difference (*P* < 0.05).

The data were means ± standard deviation (*n =* 6).

1 CON: treated with sterile saline; ETEC: treated with *E. coli*; LP-ETEC: treated with ETEC, and *L. plantarum*; LB-ETEC: treated with ETEC, and *L. brevis*.

### 3.2 Probiotics alleviate ETEC-induced intestinal injury

As shown in Fig. 1B and Table 3, compared to the CON group, piglets in the ETEC group had damaged intestinal structures (decreased villous height and increased crypt depth) with inflammatory infiltration, while piglets in the LP-ETEC and LB-ETEC groups showed reduced damage (*P* < 0.05), recovering to a level similar to the control group.

**Table 3:** Effect of two probiotics on intestinal morphology in piglets challenged with *E*. *coli*.^1^

Item	CON	ETEC	LP-ETEC	LB-ETEC
Villous height, μm	567.67 ± 34.40^a^	393.83 ± 27.05^b^	532.33 ± 27.05^a^	561.33 ± 31.18^a^
Crypt depth, μm	249.17 ± 17.58^b^	331.67 ± 12.59^a^	252.17 ± 16.58^b^	224.00 ± 18.18^b^
Villous height:crypt depth ratio	2.30 ± 0.30^a^	1.19 ± 0.11^b^	2.12 ± 0.22^a^	2.32 ± 0.28^a^

The absence of identical letter marker indicated significant difference (*P* < 0.05).

The data were means ± standard deviation (*n =* 6).

1 CON: treated with sterile saline; ETEC: treated with *E. coli*; LP-ETEC: treated with ETEC, and *L. plantarum*; LB-ETEC: treated with ETEC, and *L. brevis*.

### 3.3 Probiotics affect the composition of serum metabolites in piglets

**Fig. 2:**
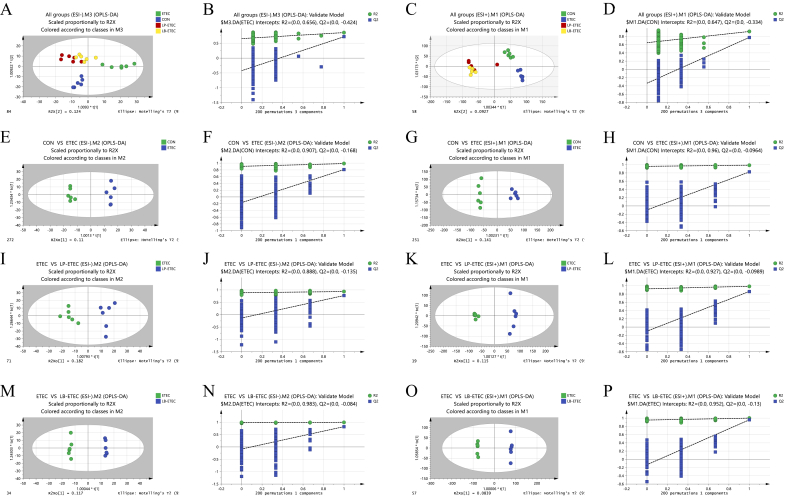
Multivariable statistical comparison plots between groups. Orthogonal Partial Least Squares Discriminant Analysis (OPLS-DA) score plot of (A) all groups (ESI-), (B) OPLS-DA score plot of all groups (ESI), (C) OPLS-DA (Validate Model) score plot of all groups (ESI-), (D) OPLS-DA (Validate Model) score plot of all groups (ESI), (E) OPLS-DA score plot of CON vs ETEC (ESI-), (F) OPLS-DA score plot of CON vs ETEC (ESI), (G) OPLS-DA (Validate Model) score plot of CON vs ETEC (ESI-), (H) OPLS-DA (Validate Model) score plot of CON vs ETEC (ESI), (I) OPLS-DA score plot of ETEC vs LP-ETEC (ESI-), (J) OPLS-DA score plot of ETEC vs LP-ETEC (ESI), (K) OPLS-DA (Validate Model) score plot of ETEC vs LP-ETEC (ESI-), (L) OPLS-DA (Validate Model) score plot of ETEC vs LP-ETEC (ESI), (M) OPLS-DA score plot of ETEC vs LB-ETEC (ESI-), (N) OPLS-DA score plot of ETEC vs LB-ETEC (ESI), (O) OPLS-DA (Validate Model) score plot of ETEC vs LB-ETEC (ESI-), (P) OPLS-DA (Validate Model) score plot of ETEC vs LB-ETEC (ESI). CON = sterile saline; ETEC = *E. coli*; LP-ETEC = ETEC and *L. plantarum*; LB-ETEC = ETEC and *L. brevis*.

**Fig. 3:**
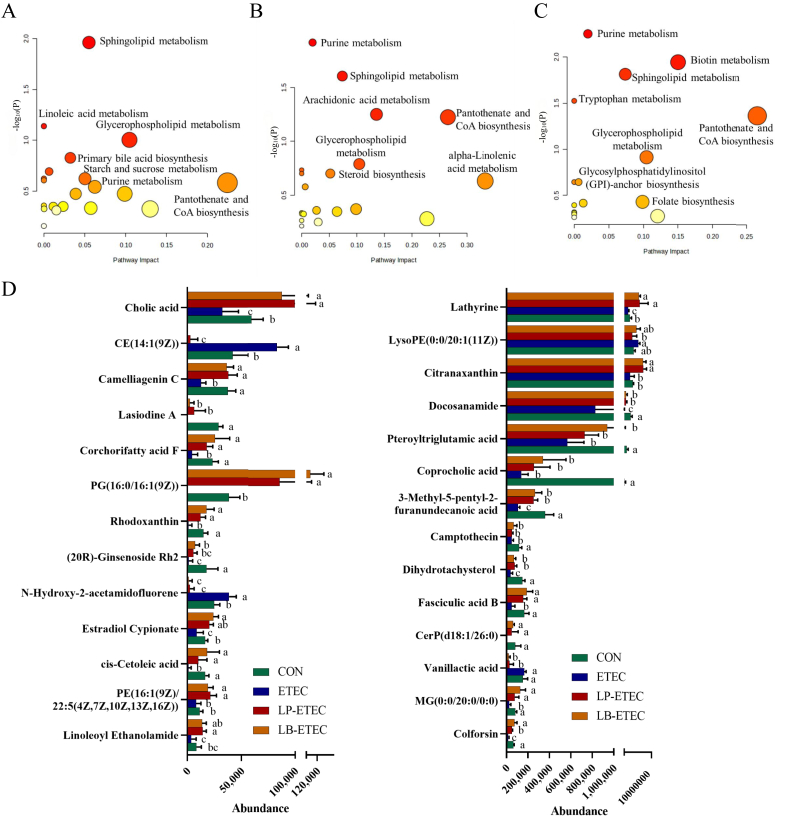
Impact of two probiotics on the metabolites of piglets. Enrichment pathways of differential metabolites between (A) CON and ETEC groups, (B) ETEC and LP-ETEC groups, (C) ETEC and LB-ETEC groups; (D) differential metabolites between four groups. The absence of identical letter marker indicated significant difference (*P* < 0.05). The data were means ± standard deviation (*n =* 6). CON: treated with sterile saline; ETEC: treated with *E. coli*; LP-ETEC: treated with ETEC and *L. plantarum*; LB-ETEC: treated with ETEC and *L. brevis*.

According to the results of principal component analysis and Orthogonal Partial Least Squares Discriminant Analysis (OPLS-DA) prediction, the similarity of metabolites in each group was extremely high, and there were great differences between groups (Fig. 2). The results of enrichment metabolic pathway analysis showed that the enriched metabolic pathways between CON and ETEC groups include sphingolipid metabolism, linoleic acid metabolism, glycerophospholipid metabolism, primary bile acid biosynthesis, starch and sucrose metabolism, purine metabolism, and pantothenate and coenzyme A biosynthesis (Fig. 3A). The functional biochemical reactions identified through metabolomics were significantly excessive, indicating their critical roles in specific biological contexts, and these pathways are referred to as significantly enriched pathways. The enriched metabolic pathways between ETEC and LP-ETEC groups include purine metabolism, sphingolipid metabolism, arachidonic acid metabolism, alpha-Linolenic acid metabolism, glycerophospholipid metabolism, pantothenate and CoA biosynthesis, and steroid biosynthesis (Fig. 3B). The enriched metabolic pathways between ETEC and LB-ETEC groups include purine metabolism, biotin metabolism, sphingolipid metabolism, tryptophan metabolism, glycerophospholipid metabolism, pantothenate and CoA biosynthesis, glycosylphosphatidylinositol (GPI)-anchor biosynthesis, and folate biosynthesis (Fig. 3C).

**Fig. 4:**
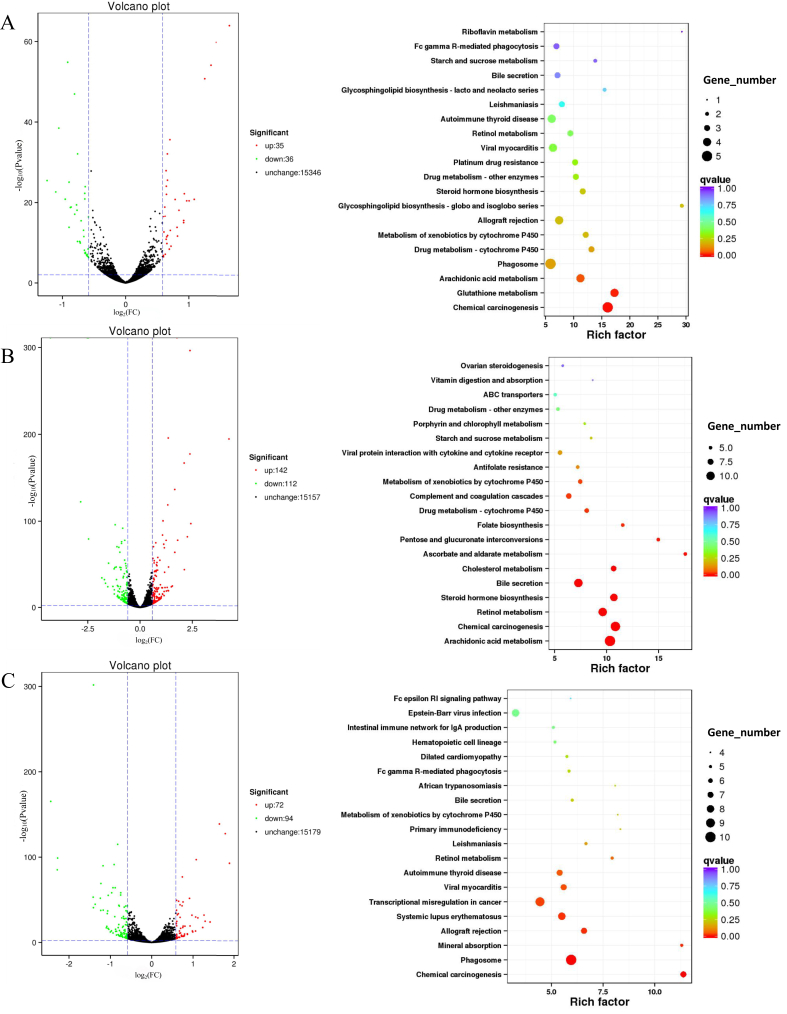
Impact of two probiotics on colonic transcriptome of piglets. Volcano map and enrichment pathways of differentially expressed genes between (A) CON and ETEC groups, (B) ETEC and LP-ETEC groups, (C) ETEC and LB-ETEC groups. CON: treated with sterile saline; ETEC: treated with *E. coli*; LP-ETEC: treated with ETEC and *L. plantarum*; LB-ETEC: treated with ETEC and *L. brevis*.

Different metabolites between four groups were also analyzed in this study (Fig. 4D). ETEC decreased the levels of phosphatidylethanolamine (PE) [16:1(9Z)/22:5(4Z, 7Z, 10Z, 13Z, 16Z)], phosphatidylglycerol (PG) [16:0/16:1(9Z)], and monoacylglyceride (MG) (0:0/20:0/0:0) in serum metabolites of piglets, while both probiotics significantly increased their levels (*P* < 0.05). Compared to the CON group, lysophosphatidylethanolamine (LysoPE) (0:0/20:1(11Z)), and cholesterol ester (CE) (14:1(9Z)) of piglets in the ETEC group were increased, while the content of these lipids was reduced to normal levels in the probiotics-treated groups. In the ETEC group, the content of cis-cetoleic acid, 3-methyl-5-pentyl-2-furanundecanoic acid, and corchorifatty acid F were notably decreased compared to those in the CON group (*P* < 0.05), while the treatment of probiotics increased these metabolites significantly (*P* < 0.05). In addition, the levels of vanillactic acid in the CON and ETEC groups were not significantly different, but both probiotics decreased their levels significantly (*P* < 0.05). 20(R)-Ginsenoside Rh2, coprocholic acid, cholic acid, estradiol cypionate, lathyrine, docosanamide, linoleoyl ethanolamide, and ceramide phosphate (CerP) (d18:1/26:0) were significantly decreased after ETEC attack (*P* < 0.05), while these metabolites were increased in LP-ETEC and LB-ETEC, some of which were increased to levels similar to those in CON group. On the contrary, N-hydroxy-2-acetamidofluorene increased significantly in the ETEC group, but decreased significantly in probiotics-treated groups. ETEC decreased the contents of rhodoxanthin, dihydrotachysterol, pteroyltriglutamic acid, citranaxanthin, colforsin, camelliagenin C, fasciculic acid B, camptothecin, and lasiodine A significantly (*P* < 0.05), while the contents of them were increased in LB-ETEC and LP-ETEC groups (*P* < 0.05).

### 3.4 Probiotics affect the RNA expression of piglets

Volcano maps and enrichment pathways were made using differentially expressed genes between different groups. There were 71 differentially expressed genes between CON and ETEC groups, which were enriched in riboflavin metabolism, starch and sucrose metabolism, bile secretion, glycosphingolipid biosynthesis—lacto and neolacto series, viral myocarditis, metabolism of xenobiotics by cytochrome P450, and glutathione metabolism (Fig. 4A). In the ETEC and LP-ETEC groups, there were 254 differentially expressed genes, which were mainly enriched in the ATP-binding cassette transporters, starch and sucrose metabolism, metabolism of xenobiotics by cytochrome P450, porphyrin and chlorophyll metabolism, folate biosynthesis, bile secretion, and steroid hormone biosynthesis (Fig. 4B). In the ETEC and LB-ETEC groups, there were 166 differentially expressed genes, which were mainly enriched in the intestinal immune network for immunoglobulin A production, hematopoietic cell lineage, Fc gamma receptors-mediated phagocytosis, primary immunodeficiency, viral myocarditis, transcriptional misregulation in cancer, systemic lupus erythematosus, and allograft rejection (Fig. 4C).

**Fig. 5:**
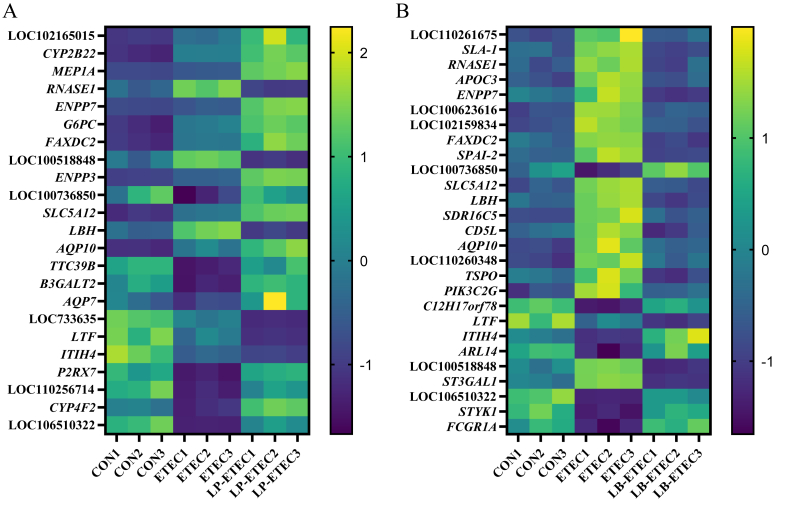
Impact of two probiotics on expressed genes of piglets. (A) Genes regulated by *L*. *plantarum*; (B) genes regulated by *L*. *brevis*. CON: treated with sterile saline; ETEC: treated with *E. coli*; LP-ETEC: treated with ETEC and *L. plantarum*; LB-ETEC: treated with ETEC and *L. brevis*. The full names of the genes are shown in Table S1.

There were 23 differentially expressed genes co-regulated by CON vs ETEC and ETEC vs LP-ETEC groups with |Log_2_ FC| greater than 1, of which *RNASE1*, LOC100518848, and *LBH* were up-regulated in the ETEC group but down-regulated in the LP-ETEC group, and LOC100736850, *TTC39B*, *B3GALT2*, *AQP7*, *P2RX7*, LOC110256714, *CYP4F2*, and LOC106510322 were down-regulated in the ETEC group but up-regulated in the LP-ETEC group (Fig. 5A). The full names of the genes are shown in Table S1. There were 27 differentially expressed genes co-regulated by CON vs ETEC and ETEC vs LB-ETEC groups with |Log_2_ FC| greater than 1, of which LOC110261675, *SLA-1*, *RNASE1*, *APOC3*, *ENPP7*, LOC100623616, LOC102159834, *FAXDC2*, *SPAI-2*, *SLC5A12*, *LBH*, *SDR16C5*, *CD5L*, *AQP10*, LOC110260348, *TSPO*, *PIK3C2G*, LOC100518848, and *ST3GAL1* were up-regulated in the ETEC group but down-regulated in the LB-ETEC group, and LOC100736850, *C12H17orf78*, *ITIH4*, *ARL14*, LOC106510322, *STYK1*, and *FCGR1A* were down-regulated in the ETEC group but up-regulated in the LB-ETEC group (Fig. 5B).

**Fig. 6:**
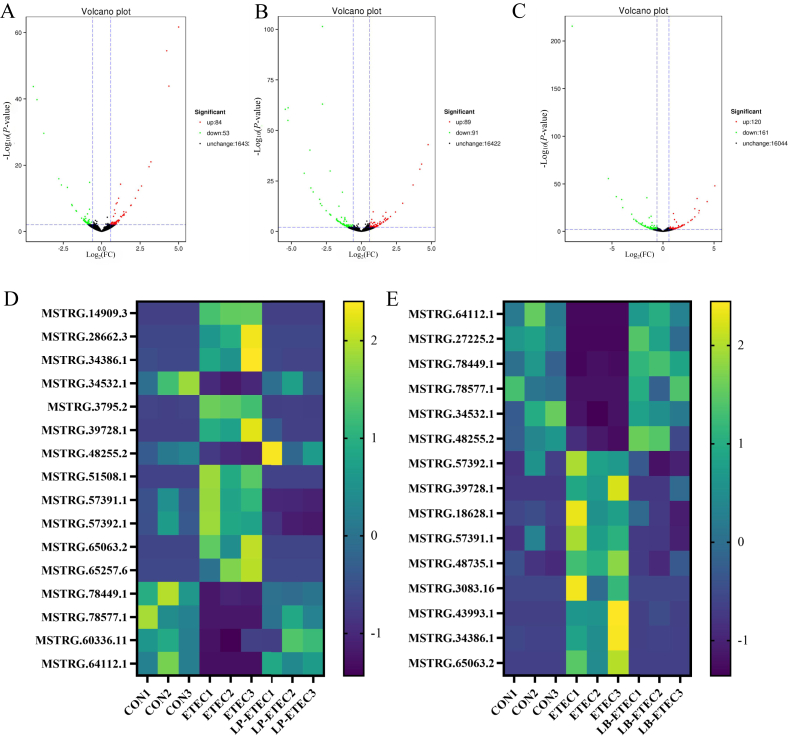
Impact of two probiotics on lncRNAs of piglets. Volcano map of different lncRNAs between (A) CON and ETEC groups, (B) ETEC and LP-ETEC groups, (C) ETEC and LB-ETEC groups; (D) lncRNAs regulated by *L*. *plantarum*; (E) lncRNAs regulated by *L*. *brevis*. CON: treated with sterile saline; ETEC: treated with *E. coli*; LP-ETEC: treated with ETEC and *L. plantarum*; LB-ETEC: treated with ETEC and *L. brevis*. lncRNAs = long non-coding RNAs.

Volcano maps of different long non-coding RNAs (lncRNAs) between different groups were also made. There were 137 different lncRNA between the CON and ETEC groups (Fig. 6A), 180 different lncRNA between ETEC and LP-ETEC groups (Figs. 6B), 281 different lncRNA between ETEC and LB-ETEC groups (Fig. 6C). There were 16 different lncRNA co-regulated by CON vs ETEC and ETEC vs LP-ETEC groups with |Log_2_ FC| greater than 1, of which MSTRG.14909.3, MSTRG.28662.3, MSTRG.34386.1, MSTRG.3795.2, MSTRG.39728.1, MSTRG.51508.1, MSTRG.57391.1, MSTRG.57392.1, MSTRG.65063.2, and MSTRG.65257.6 were up-regulated in the ETEC group but down-regulated in the LP-ETEC group, and MSTRG.34532.1, MSTRG.48255.2, MSTRG.78449.1, MSTRG.78577.1, MSTRG.60336.11, and MSTRG.64112.1 were down-regulated in the ETEC group but up-regulated in the LP-ETEC group (Fig. 6D). There were 15 different lncRNA co-regulated by CON vs ETEC and ETEC vs LB-ETEC groups with |Log_2_ FC| greater than 1, of which MSTRG.57392.1, MSTRG.39728.1, MSTRG.18628.1, MSTRG.57391.1, MSTRG.48735.1, MSTRG.3083.16, MSTRG.43993.1, MSTRG.34386.1, and MSTRG.65063.2 were up-regulated in the ETEC group but down-regulated in the LB-ETEC group, and MSTRG.64112.1, MSTRG.27225.2, MSTRG.78449.1, MSTRG.78577.1, MSTRG.34532.1, and MSTRG.48255.2 were down-regulated in the ETEC group but up-regulated in the LB-ETEC group (Fig. 6E).

According to the interaction between lncRNA and its target genes, the interaction between co-regulated lncRNA and co-regulated target genes is the potential mechanism for probiotics to alleviate piglet diarrhea. *L*. *plantarum* may alleviate diarrhea symptoms in piglets by regulating lncRNA MSTRG.3795.2 and its target genes *LBH* and *RNASE1*, MSTRG.39728.1 and gene *RNASE1*, MSTRG.51508.1 and genes *P2RX7*, *B3GALT2*, and LOC110256714, MSTRG.64112.1 and genes *LBH* and *RNASE1*, MSTRG.65063.2 and genes *RNASE1* and *LBH* (Table 4). *L*. *brevis* may alleviate diarrhea symptoms in piglets by regulating lncRNA MSTRG.64112.1 and genes *LBH* and *RNASE1*, MSTRG.78449.1 and genes *CD5L*, *SPAI-2*, and *ST3GAL1*, MSTRG.39728.1 and gene *RNASE1*, MSTRG.18628.1 and gene *ST3GAL1*, MSTRG.48735.1 and genes LOC100623616, *SLC5A12*, LOC102159834, *APOC3*, LOC110261675, *AQP10*, *PIK3C2G*, *FCGR1A*, *FAXDC2*, and *SDR16C5*, MSTRG.34386.1 and gene *SLA-1*, MSTRG.65063.2 and genes *RNASE1* and *LBH* (Table 5).

**Table 4:** Potential interaction between lncRNA and mRNA regulated by *L*. *plantarum*.^1^

LncRNA	Log_2_ FC (CON vs ETEC)	Regulated (CON vs ETEC)	Log_2_ FC (ETEC vs LP-ETEC)	Regulated (ETEC vs LP-ETEC)	Target gene	Log_2_ FC (CON vs ETEC)	Regulated (CON vs ETEC)	Log_2_ FC (ETEC vs LP-ETEC)	Regulated (ETEC vs LP-ETEC)
MSTRG.3795.2	4.228813	up	−5.22837	down	*LBH*	0.66198	up	−0.96547	down
MSTRG.3795.2	4.228813	up	−5.22837	down	*RNASE1*	0.690292	up	−1.54286	down
MSTRG.39728.1	1.065023	up	−1.02807	down	*RNASE1*	0.690292	up	−1.54286	down
MSTRG.51508.1	3.081889	up	−3.4383	down	*P2RX7*	−0.64024	down	0.691884	up
MSTRG.51508.1	3.081889	up	−3.4383	down	*B3GALT2*	−0.86301	down	1.174467	up
MSTRG.51508.1	3.081889	up	−3.4383	down	LOC110256714	−0.63853	down	0.638785	up
MSTRG.64112.1	−4.20457	down	4.761752	up	*LBH*	0.66198	up	−0.96547	down
MSTRG.64112.1	−4.20457	down	4.761752	up	*RNASE1*	0.690292	up	−1.54286	down
MSTRG.65063.2	3.201181	up	−3.60758	down	*RNASE1*	0.690292	up	−1.54286	down
MSTRG.65063.2	3.201181	up	−3.60758	down	*LBH*	0.66198	up	−0.96547	down

lncRNA = long non-coding RNA; FC = fold change.

1 CON: treated with sterile saline; ETEC: treated with *E. coli*; LP-ETEC: treated with ETEC, and *L. plantarum*.

**Table 5:** Potential interaction between lncRNA and mRNA regulated by *L*. *brevis*.^1^

LncRNA	Log_2_ FC (CON vs ETEC)	Regulated (CON vs ETEC)	Log_2_ FC (ETEC vs LB-ETEC)	Regulated (ETEC vs LB-ETEC)	Target gene	Log_2_ FC (CON vs ETEC)	Regulated (CON vs ETEC)	Log_2_ FC (ETEC vs LB-ETEC)	Regulated (ETEC vs LB-ETEC)
MSTRG.64112.1	−4.20456741	down	5.080362	up	*LBH*	0.66198	up	−0.93645	down
MSTRG.64112.1	−4.20456741	down	5.080362	up	*RNASE1*	0.690292	up	−0.85233	down
MSTRG.78449.1	−2.228658999	down	3.34284	up	*CD5L*	0.593419	up	−0.93543	down
MSTRG.78449.1	−2.228658999	down	3.34284	up	*SPAI-2*	1.438468	up	−2.44159	down
MSTRG.78449.1	−2.228658999	down	3.34284	up	*ST3GAL1*	0.660341	up	−1.4077	down
MSTRG.39728.1	1.06502306	up	−1.00999	down	*RNASE1*	0.690292	up	−0.85233	down
MSTRG.18628.1	1.099655938	up	−1.07541	down	*ST3GAL1*	0.660341	up	−1.4077	down
MSTRG.48735.1	1.2400151	up	−1.23415	down	LOC100623616	1.251631	up	−1.17607	down
MSTRG.48735.1	1.2400151	up	−1.23415	down	*SLC5A12*	0.643601	up	−0.6646	down
MSTRG.48735.1	1.2400151	up	−1.23415	down	LOC102159834	0.717361	up	−0.76085	down
MSTRG.48735.1	1.2400151	up	−1.23415	down	*APOC3*	0.639998	up	−0.58755	down
MSTRG.48735.1	1.2400151	up	−1.23415	down	LOC110261675	0.928392	up	−0.80996	down
MSTRG.48735.1	1.2400151	up	−1.23415	down	*AQP10*	1.654498	up	−1.17134	down
MSTRG.48735.1	1.2400151	up	−1.23415	down	*PIK3C2G*	0.917408	up	−0.87406	down
MSTRG.48735.1	1.2400151	up	−1.23415	down	*FCGR1A*	−0.60763	down	0.861348	up
MSTRG.48735.1	1.2400151	up	−1.23415	down	*FAXDC2*	0.783096	up	−1.24221	down
MSTRG.48735.1	1.2400151	up	−1.23415	down	*SDR16C5*	1.003659	up	−0.87212	down
MSTRG.34386.1	2.386494606	up	−3.068	down	*SLA-1*	0.700443	up	−0.82276	down
MSTRG.65063.2	3.201181316	up	−3.94159	down	*RNASE1*	0.690292	up	−0.85233	down
MSTRG.65063.2	3.201181316	up	−3.94159	down	*LBH*	0.66198	up	−0.93645	down

lncRNA = long non-coding RNA; FC = fold change.

1 CON: treated with sterile saline; ETEC: treated with *E. coli*; LB-ETEC: treated with ETEC, and *L. brevis*.

### 3.5 Probiotics regulate gut microbiota in piglets

**Fig. 7:**
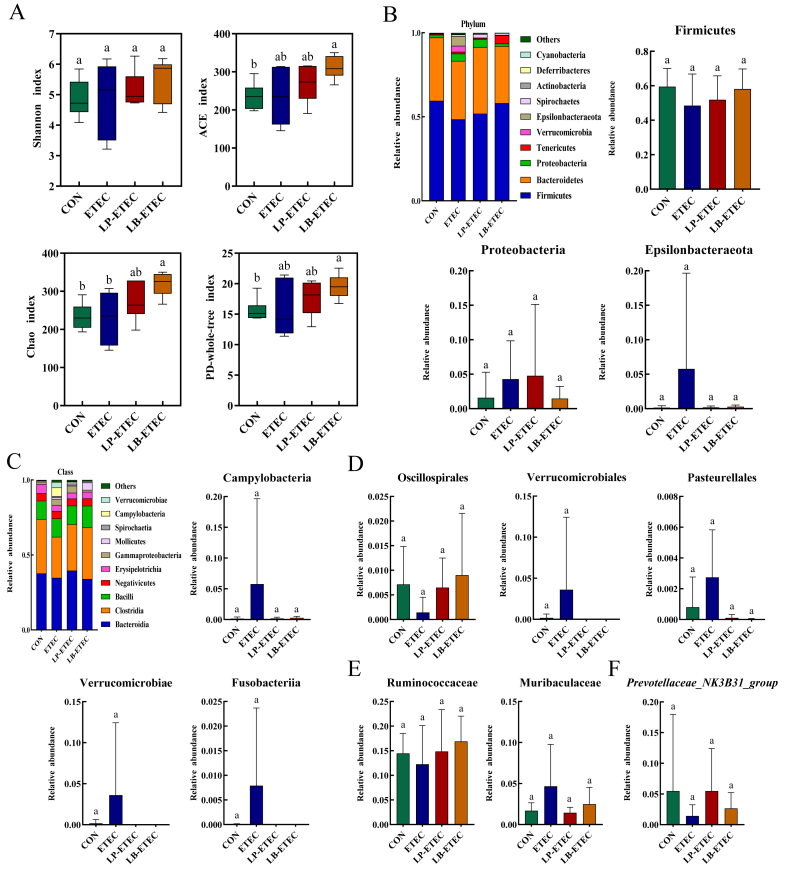
Impact of two probiotics on the microflora of piglets. (A) Gut microbial diversity; (B) microflora at the phylum level; (C) microflora at the class level; (D) microflora at the order level; (E) microflora at the family level; (F) microflora at the genus level CON group. The absence of identical letter marker indicated a significant difference (*P* < 0.05). The data were means ± standard deviation (*n =* 6). CON: treated with sterile saline; ETEC: treated with *E. coli*; LP-ETEC: treated with ETEC and *L. plantarum*; LB-ETEC: treated with ETEC and *L. brevis*.

As shown in Fig. 7A, there was no significant difference in the Shannon index, ACE (accumulated cyclone energy) index, Chao index, and Phylogenetic Diversity-whole-tree index of piglets between the ETEC group and the CON group. However, after *L. brevis* treatment, the Chao index of piglets increased significantly (*P* < 0.05), while the ACE index and PD-whole-tree index also tended to increase.

At the phylum level, 13 microorganisms were present in each group, of which Firmicutes, Bacteroidetes, and Proteobacteria exceeded 87% of total microorganisms (Fig. 7B). In the four groups, Firmicutes accounted for 59.5288%, 48.5306%, 51.888%, and 58.1389%, respectively. The abundance of Bacteroidetes was 37.6734%, 34.7675%, 39.5676% and 33.9831%, respectively. Proteobacteria accounted for 1.5838%, 4.2961%, 4.7792% and 1.4721%, respectively. After the ETEC attack, the piglets showed a decrease in the abundance of Firmicutes, while two probiotics slightly increased the abundance of it. In contrast, the ETEC group showed an increasing trend in the abundance of Proteobacteria and Epsilonbacteraeota compared to the CON group, while the probiotics slightly decreased the abundance of both bacteria.

At the class level, Bacteroidia, Bacilli, and Clostridia exceeded 70% of total microorganisms (Fig. 7C). In the four groups, Bacteroidia accounted for 37.6734%, 34.7675%, 39.5676% and 33.9831%, respectively. The abundance of Clostridia was 36.1778%, 27.2842%, 30.9419%, and 34.2903%, respectively. And Bacilli accounted for 12.2861%, 12.1823%, 12.3207% and 14.4941%, respectively. There was a tendency for ETEC to increase the abundance of Campylobacteria, Verrucomicrobiota, and Fusobacteria compared to the CON group, while both the probiotics slightly decreased the abundance of these bacteria.

At the order level, ETEC slightly reduced the abundance of Oscillospirales, while *L. plantarum* and *L. brevis* tended to increase its abundance (Fig. 7D). On the contrary, ETEC exposure was associated with a slight increase in the abundance of Verrucomicrobiales and Pasteurellales, while these two probiotics slightly reduced their abundance. At the family level, ETEC slightly reduced the abundance of Ruminococcaceae, while *L. plantarum and L. brevis* tended to increase its abundance (Fig. 7E). On the contrary, ETEC slightly improved the growth of Muribaculaceae, while probiotics slightly reduced its abundance. In addition, the abundance of Prevotellaceae*_NK3B31_group* was slightly reduced by ETEC, with *L. plantarum and L. brevis* tended to increase its abundance (Fig. 7F).

## 4 Discussion

Lactic acid bacteria have been widely used in the treatment of diseases, and the addition of lactic acid bacteria to feeds can significantly reduce pro-inflammatory factors and enhance the body's immunity (Garbacz, 2022; Han et al., 2021; Saha and Saroj, 2022). In this study, the effects of two probiotics on damage induced by ETEC in piglets were investigated. Consistent with prior research (Ma et al., 2021), these two probiotics inhibited weight loss in piglets caused by ETEC and prevented morphological damage to the jejunum. Microbial sequencing showed that these two probiotics helped maintain the normal microbial community of piglets, and alleviated the abundance of intestinal pathogens. Serum metabolomic analysis showed an increase in the production of beneficial metabolites, including phosphatidylethanolamine and rhodoxanthin. Transcriptome analysis identified potential targets such as *LBH*, *RNASE1*, *P2RX7*, and *B3GALT2* for *L. plantarum*, and *LBH*, *RNASE1*, *CD5L*, *SPAI-2*, *ST3GAL1*, *SLC5A12*, *APOC3*, *AQP10*, *PIK3C2G*, *FCGR1A*, *FAXDC2*, *SDR16C5*, and *SLA-1* for *L. brevis*, with associated lncRNAs playing a role in the mechanism.

When an animal is injured, its liver sends a signal to the gut and reshape the microbial community, resulting in changes in the abundance of various microorganisms in the gut (Albillos et al., 2020). In this study, the abundance of Epsilonproteobacteria and Proteobacteria was increased in the intestine of ETEC-treated piglets. Proteobacteria are the intestinal microorganisms most closely associated with disease, and they have been found to be strongly linked to intestinal inflammation and metabolic diseases (Cuesta et al., 2022). Epsilonproteobacteria are harmful bacteria in some species that may cause gastrointestinal diseases. When it enters the body of animals, it usually leads to tissue infections and intestinal diseases (Vandenberg et al., 2013). *L. plantarum* and *L. brevis* can reduce the abundance of both bacteria and avoid intestinal diseases in piglets. Campylobacteria is one of the four causes of diarrheal diseases and is the most common cause of gastroenteritis from a bacterial perspective. Verrucomicrobiae flora are enriched in the mucus layer of the intestine and are present in large numbers in the human intestine, where they can have an impact on host nutritional metabolism and organism immunity. Fusobacteria is associated with the development and progression of colorectal cancer (Bum et al., 2021). Pasteurellales belong to the γ-proteobacteria and can cause human diseases. In this study, the abundance of all these bacteria was extremely low in the CON group, and ETEC increased the abundance of these bacteria, while both the probiotics restored them to normal levels. The results indicated that both probiotics can control pathogenetic microorganisms, thus protecting the intestinal health of piglets.

Recent advances in metabolomics have identified glycerophospholipids as pivotal biomarkers due to their structural and regulatory roles in biofilms, bile composition, and cell signaling. According to the results of this study, the glycerophospholipid metabolic pathway was enriched in different groups. Key glycerophospholipid metabolites, including PE, PG, LysoPE, and CE, were dynamically altered. PE, which is essential for membrane protein folding and autophagy initiation (Pohl and Jovanovic, 2019), was elevated by both probiotics, potentially enhancing membrane transporter functionality. PG is a lipid that plays a crucial role in cell physiology and it is able to reduce tumor necrosis factor-α production in lipopolysaccharide-stimulated mouse in vitro (Klein et al., 2020). Our study found that PG (16:0/16:1(9Z)) was almost absent in piglets of the ETEC group, while probiotics significantly increased their levels and contributed to the function of the immune system in piglets. Pathological LysoPE and CE levels, associated with atherosclerosis (Tsukahara et al., 2017) and foam cell formation (Sekiya et al., 2011) were exacerbated by ETEC but mitigated by probiotics, indicating therapeutic modulation of lipid homeostasis. Long-chain fatty acids (e.g., Oleic acid and corchorifatty acid F) and furan fatty acid (e.g., 3-methyl-5-pentyl-2-furanundecanoic acid) are vital for energy metabolism and antioxidant defense (Lauvai et al., 2019; Nakamura et al., 2014). These fatty acids were suppressed by ETEC but upregulated with probiotics, indicating their ability to regulate energy metabolism and antioxidant capacity in piglets. Anti-inflammatory metabolites like linoleoyl ethanolamide and CerP were depleted during ETEC infection but normalized post-probiotic administration, underscoring their role in mitigating inflammation (Berwick et al., 2019; Ishida et al., 2013). Notably, probiotics reduced levels of the carcinogen N-hydroxy-2-acetamidofluorene (Bankole et al., 2021), enhancing host safety. Vitamins (rhodoxanthin and dihydrotachysterol) and terpenoids (colforsin and fasciculic acid B), with antioxidant and anti-inflammatory properties, were significantly elevated, supporting systemic health (Dumitraş et al., 2022; Takahashi et al., 1989; Yokochi et al., 2010). Most notably, both probiotics reduced the production of pro-inflammatory metabolites by inhibiting pathogenic bacteria, and promoting the metabolism of short-chain fatty acids by symbiotic bacteria, activating host G protein-coupled receptors, and enhancing intestinal barrier function. In addition, the recovery of bile acid levels is related to the bile acid metabolism activity of symbiotic bacteria such as Clostridia, which may regulate host lipid metabolism through the Farnesoid X receptor (FXR) signaling pathway.

**Fig. 8:**
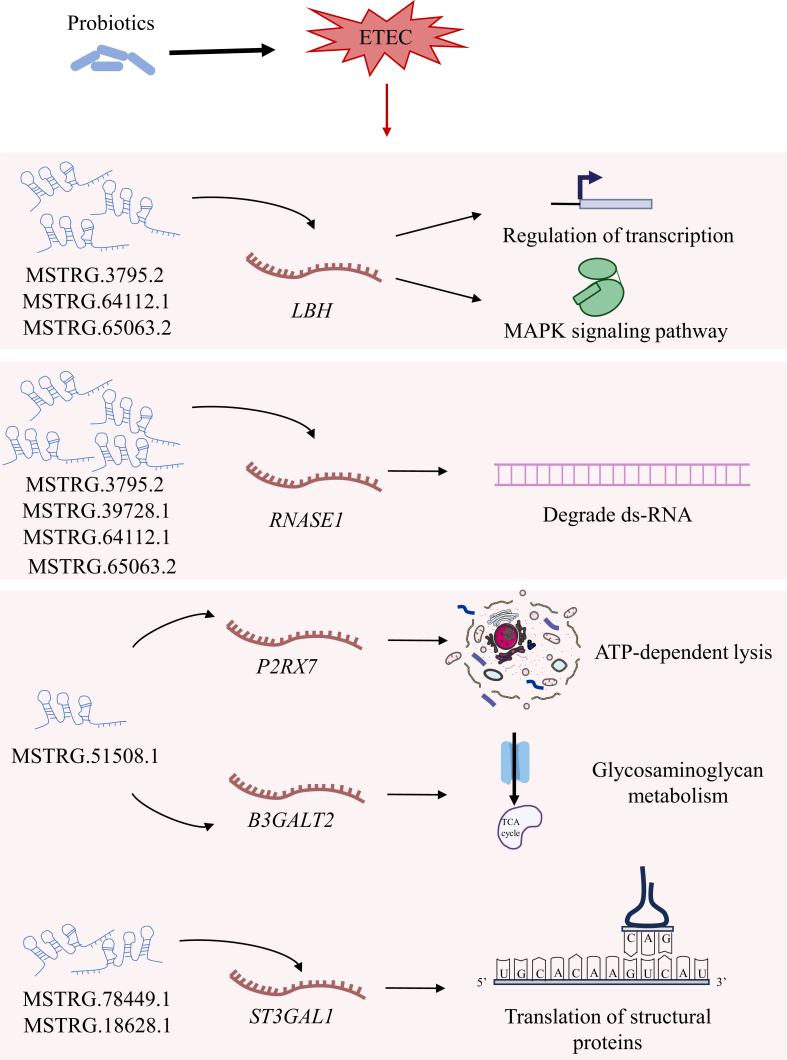
Probiotics may alleviate piglet diarrhea by regulating the expression of lncRNAs and genes. lncRNAs = long non-coding RNAs. The full names of the genes are shown in Table S1.

Among the differentially expressed genes regulated by probiotics, the conserved domain *LBH* of *LBHD1* is associated with a variety of diseases including nasopharyngeal carcinoma, rheumatoid arthritis, congenital heart disease, and human breast cancer, and is a transcriptional activator in the mitogen-activated protein kinase (MAPK) signaling pathway (Dong et al., 2019). The purinergic receptor *P2RX7* is a ligand-gated ion channel that recognizes extracellular ATP. Its extracellular ATP sensing to CD8 T cells is necessary for its ability to effectively eliminate tumors by promoting mitochondrial adaptation and has the potential to enhance tumor immunotherapy (Wanhainen et al., 2022). *B3GALT2* may play a beneficial role in neuronal survival in penumbra by regulating the Reelin pathway (Jia et al., 2021). ETEC increased the expression of *LBHD1* and decreased that of *P2RX7* and *B3GALT2* in this study, while both the probiotics restored their expression, which had a beneficial effect on the body of piglets. *CD5L* encodes soluble protein secreted by macrophages during the inflammatory response (Sanchez-Moral et al., 2023), and it is an important factor leading to adaptive resistance after anti-angiogenesis therapy (LaFargue et al., 2023). *St3gal1* is a key negative regulator of Chimeric Antigen Receptor (CAR)-T cell cancer-specific migration and promotes melanoma invasion (Hong et al., 2023b; Pietrobono et al., 2020). Apo C-III is an amino acid protein circulating with chylomicrons and other lipoproteins, it can interfere with lipoprotein Lipase (LPL) activity and promote the secretion of triglyceride (TG)-rich lipoproteins, making it a promising therapeutic target for chylomicronemia (Hegele, 2022). *Sdr16c5* is essential for embryonic and adult tissue differentiation, development, and apoptosis. It also participates in immune responses, and retinol metabolism pathways, regulates energy metabolism, and is associated with tumor formation (Hong et al., 2023a). *L. brevis* exerts beneficial effects on piglets by regulating these genes. Significantly, lncRNA is vital in many biological activities and is an important regulator of gene expression (Zhang et al., 2019). The lncRNA regulating the above target genes in this study is also part of the potential mechanism for probiotics to alleviate piglet diarrhea (Fig. 8).

It is worth noting that vitamin D_3_ may regulate the expression of antimicrobial peptide (such as β-defensins) genes and inhibit the proliferation of pathogens by binding to vitamin D receptor (VDR). Rhodoxanthin, as an antioxidant, may reduce the expression of oxidative stress-related genes (such as *HO-1*) through the Nrf2 pathway. The MAPK signaling pathway (*LBH*, *P2RX7*) and lipid metabolism pathway (*APOC3*, *SDR16C5*) were enriched in all three omics data, indicating that probiotics alleviate ETEC injury through cross omics regulation. LncRNAs (such as MSTRG. 65063.2) may serve as “molecular bridges” connecting changes in microbial communities (such as Proteobacteria) with the expression regulation of host genes (such as *B3GALT2*).

## 5 Conclusion

The results showed that *L. plantarum* and *L. brevis* had protective effects against ETEC-induced injury in piglets. They can inhibit the reduction in daily weight gain, prevent morphological damage of the jejunum, and significantly increase the content of beneficial metabolites, thereby enhancing immune defense mechanisms. In addition, they may alleviate piglet diarrhea, reduce intestinal damage, and promote immune function by regulating genes such as *LBH* and *RNASE1*.

## Credit Author Statement

**Xuebing Han:** Writing – review & editing, Writing – original draft, Methodology. **Rong Gao:** Writing – review & editing, Investigation. **Sujuan Ding:** Writing – review & editing. **Hao Yao:** Supervision. **Jun Fang:** Project administration. **Gang Liu:** Supervision, Funding acquisition.

## Declaration of competing interest

We declare that we have no financial and personal relationships with other people or organizations that can inappropriately influence our work, and there is no professional or other personal interest of any nature or kind in any product, service and/or company that could be construed as influencing the content of this paper.
