# Supplementary material for: Lactobacillus plantarum and Lactobacillus brevis alleviate enterotoxigenic Escherichia coli-induced intestinal inflammation and stabilize intestinal microorganisms and serum metabolites in piglets
Source: Anim Nutr. 2025 Jun 20;22:165–78. doi: 10.1016/j.aninu.2025.04.005 (PMC12391826; doi:10.1016/j.aninu.2025.04.005)
Supplement: Multimedia component 1 [file mmc1.docx]

Table S1 Full names of genes

| Abbreviation | Full name of gene |
| --- | --- |
| *APOC3* | Apolipoprotein C-Ⅲ |
| *AQP10* | Aquaglyceroporin-10 |
| *ARL14* | ADP-ribosylation factor like GTPase 14 |
| *AQP7* | Aquaporin 7 |
| *CD5L* | CD5 molecule like |
| *CYP4F2* | Cytochrome P450 family 4 subfamily F member 2 |
| *C12H17orf78* | Chromosome 12 C17orf78 homolog |
| *ENPP7* | Ectonucleotide pyrophosphatase/phosphodiesterase 7 |
| *FAXDC2* | Fatty acid hydroxylase domain-containing protein 2 |
| *FCGR1A* | Fc fragment of IgG, high affinity Ia, receptor |
| *HO-1* | Heme oxygenase-1 |
| *ITIH4* | Inter-alpha-trypsin inhibitor heavy chain 4 |
| *LBH* | Limb bud and heart development homolog |
| *LBHD1* | LBH domain containing 1 |
| *NRF2* | Nuclear factor erythroid 2-related factor 2 |
| *PIK3C2G* | Phosphatidylinositol-4-phosphate 3-kinase catalytic subunit type 2 gamma |
| *P2RX7* | Purinergic receptor P2X, ligand-gated Ion channel, 7 |
| *RNASE1* | Ribonuclease, RNase a family, 1 |
| *SDR16C5* | Short chain dehydrogenase/reductase family 16C member 5 |
| *SLA-1* | Swine leukocyte antigen-1 |
| *SLC5A12* | Solute carrier family 5 (Sodium/Monocarboxylate cotransporter), member 12 |
| *SPAI-2* | Sodium/potassium ATPase inhibitor SPAI-2 |
| *STYK1* | Serine/threonine/tyrosine kinase 1 |
| *ST3GAL1* | ST3 beta-galactoside alpha-2,3-sialyltransferase 1 |
| *TTC39B* | Tetratricopeptide repeat domain 39B |
| *TSPO* | Translocator protein |
